# Seroprevalence and Associated Risk Factors of Infectious Bronchitis Virus in Chicken in Northwest Ethiopia

**DOI:** 10.1155/2021/4553890

**Published:** 2021-11-13

**Authors:** M. Birhan, M. Temesgen, A. Shite, N. Berhane, M. Bitew, E. Gelaye, T. Abayneh, B. Getachew

**Affiliations:** ^1^Institute of Biotechnology, University of Gondar, Gondar, Ethiopia; ^2^College of Veterinary Medicine and Animals Sciences, Gondar, Ethiopia; ^3^Ethiopian Biotechnology Institute, Addis Ababa, Ethiopia; ^4^National Veterinary Institute, Bishoftu, Ethiopia

## Abstract

Avian infectious bronchitis virus is a highly contagious disease occurring in respiratory, urogenital, and reproductive tissues of chicken causing considerable losses due to death, egg drop, and reduced production. This preliminary study was conducted to investigate the prevalence of antibodies against infectious bronchitis virus (IBV) and to assess the potential risk factors in chickens of northwest Ethiopia. A cross-sectional study was conducted from November 2020 to June 2021. A total of 768 serum samples from three zones were collected. To investigate the presence of antibodies against IBV, the indirect ELISA serological test was applied. Positivity for anti-IBV antibodies was observed in 23.96% (95% CI: 20.98–27.14) of the samples. The mixed-effect logistic regression analysis of potential risk factors showed that IBV prevalence was significantly higher in young chickens than adults (*p* < 0.001) and higher in intensive farm type than in extensive type (*p* < 0.001). Based on the production purposes of the chickens, the odds of seropositivity for IB was significantly higher in layers than in broilers (*p* < 0.001) and dual purposes (*p* < 0.001). This study revealed higher seroprevalence in farms which had the “all-in-all-out” rearing method than in farms with different batches in one house with a significant difference (*p* < 0.001), higher seroprevalence in the poor ventilated type than in good ones (*p* < 0.001), and higher seroprevalence in the houses that did not remove used litter at all than houses of completely disposed and partially disposed litter (*p*=0.002). Moreover, disinfection of houses had significant effect on the occurrence of IB. Having personal protective equipment was significantly affecting the occurrence of IB, being higher in the farms that have no wearing clothes and shoe than in those having wearing clothes and shoe (*p*=0.002). In conclusion, the seroprevalence finding in the present study indicated that the organism is circulating among the population of chickens and high enough to cause significant economic losses Therefore, poultry houses should be cleaned, disinfected, and well ventilated and farm attendants should have separate farm shoe and clothes. Further studies on the virus isolation and molecular characterization of the target gene are needed in the study area.

## 1. Introduction

Ethiopia is gifted with numerous livestock species with an estimated population of 56.5 million poultry [1]. Poultry production has an important economic, social, and cultural benefit and plays a significant role in family nutrition in developing countries including Ethiopia. The proportional contribution of poultry to the total animal protein production of the world is believed to increase by 40%, the major increase being in the developing world [2]. However, there are still challenges that are continually faced by poultry farms and farmers. For example, disease and predators, extension problems, exotic chicken adaptation challenges, and veterinary service shortages are the major poultry sector headaches [3].

High morbidity rate and reduced egg and meat production are caused by respiratory tract infections, which are of paramount importance in the poultry industry because high mortality may also occur in poorly managed cases [4]. Several viruses have been associated with respiratory infection in poultry, such as Newcastle disease, Infectious bronchitis virus, infectious laryngotracheitis (ILT), pneumoviral infections, and avian influenza which are among the diseases that could jeopardize the health status of the poultry [5]. These respiratory pathogens are of major importance because they can cause disease independently and self-sufficiently, in association with each other or in association with bacterial or some of the risk factors like environmental factors [4].

Infectious bronchitis is the most important and major viral disease of poultry among those that causes respiratory infections. It is a severe and acute disease of poultry caused by the infectious bronchitis virus (IBV) [6]. IBV is a single-stranded positive sense, enveloped ribonucleic acid (RNA) virus of 27–28 kb length [7, 8]. Its taxonomic classification is order Nidovirales, family Coronaviridae, genus *Gammacoronavirus*, and species *Avian coronavirus* [9]. The viral genome is made up of structural and nonstructural protein-coding gene segments [10].

The infectious bronchitis virus is communicated through the airborne, mechanical transmission between chickens, houses, and farms [11]. Airborne transmission mainly occurs through aerosol, in droplets expelled during coughing or sneezing by the infected. Spread of the disease through a flock is very rapid [12]. And, mechanical transmission occurs by personal contact and material and equipment sharing between the farm and flocks [13].

This disease spread worldwide since its first acknowledgment in 1941 in the USA and basically affects the respiratory tract, kidneys, and reproductive system causing respiratory distress, kidney damage, and decrease in egg production [14]. More importantly, it causes significant economic losses to poultry producers [15]. It may result a morbidity and mortality of up to 80 and 20%, respectively [16]. However, mortality may increase up to 50% with some strains that cause nephritis or when opportunistic pathogens such as *Escherichia coli* complicate the disease [17]. Obviously, losses from production inefficiencies are more than mortality. In broilers, infectious bronchitis leads to poor weight gain and loss of profit at slaughter. In layers, it causes loss of egg qualities and egg production may drop down to 10–50% [18]. When young pullets are affected, damage to the reproductive tract can result in layers and breeders failing to come into production [17].

Diagnosis of IBV can be achieved by a number of tests including agar gel precipitation test, hemagglutination inhibition, and ELISA [19]. Nowadays, reverse transcriptase polymerase chain reaction (RT-PCR) and/or RT-LAMP are becoming more preferred than others for the diagnosis of this disease, because of its sensitivity and specificity [13].

All-in-all-out operations of rearing along with good biosafety measures form the basis of prevention. However, vaccination forms the backbone of IB control programme; both live and inactivated vaccines are available. However, vaccination is less fruitful as a result of persistent rise of antigenic variance and less cross protection between the variance. Since the disease continues to emerge with diversity, it is much difficult to control infection as no specific treatment is available [13].

### 1.1. Statement of the Problem and Justification

To the best of the authors' knowledge, till now, few studies have been conducted so far in Ethiopia. Hutton et al. [20] revealed the presence of the 793B genotype in commercial chicken farms with high (94.5%) seroprevalence, from the Ethiopian Institute of Agricultural Research (EIAR), Debre Zeit, Ethiopia. Tesfaye et al. [21] also reported M41, D-274, 793B, and QX serotypes, with a prevalence of 74.88% and 68.75% in the unvaccinated backyard and commercial farms, respectively. Moreover, Tegegne et al. [22] detected IBV 793B (GI-13) strains, with a prevalence of 6% from intensive and backyard unvaccinated flocks of the Jimma Zone. There was no study in northwest Ethiopia.

Despite reports of respiratory diseases in chicken from all production systems, there is dearth of information available on infectious bronchitis (IB) and IBV [23]. There are observed pieces of evidence suggesting the occurrence of IB in the field. In the absence of official reports or published research studies, there is no evidence of circulation of the IBV in the northwest part of Ethiopia. Moreover, it is not a well-studied disease in Ethiopia probably because it is not commonly encountered and more especially it is usually masked by other infections like Newcastle disease and other respiratory diseases.

Furthermore, there is lack of effective treatment for infectious bronchitis; the best option for poultry farmers and veterinarians is prevention through biosecurity protocols and vaccination schedules aligned with the local reality [24]. Different types of vaccines are used for the protection of infectious bronchitis, but not all of them are available or authorized in every country [25]. On the other hand, estimating the seroprevalence of IB and identifying associated risk factors of infectious bronchitis are important to plan effective prevention and control measures. Therefore, the objectives of this study were to estimate the seroprevalence of IB and identify associated risk factors in chickens in northwest Ethiopia.

## 2. Materials and Methods

### 2.1. Study Area Description

**Figure 1:**
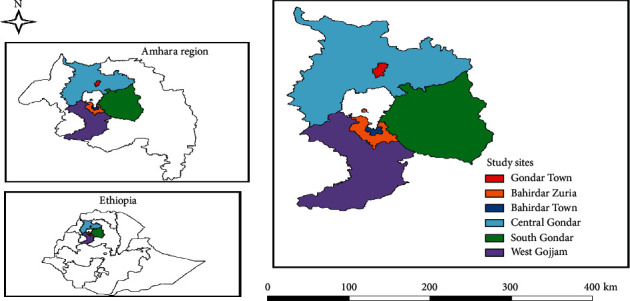
Map of the study area.

This study was conducted in the Amhara national regional state (ANRS), at three selected zones of northwest Ethiopia (Figure 1). The ANRS is located in the northwestern part of Ethiopia between 9°20′ and 14°20′ N latitude and 36°20′ and 40°20′ E longitude. The study area has diverse agroclimatic conditions; ranging from hot lowlands to cold highlands [26]. Of the 56.5 million national poultry population, about 31% are in the Amhara region and contribute to about 28% of the total annual national egg and poultry meat production. South Gondar (1.98 million), Central Gondar, and West Gojjam (3.436 million) contribute to the major proportion of the poultry population in the Amhara region [27].

The Central Gondar zone is found in ANRS, and its capital city, Gondar, is located 727 km away from Addis Ababa, the capital city of Ethiopia. It was a part of North Gondar. According to Birhan et al. [28], the Central Gondar zone is located geographically between coordinates 12.3° to 13.38° N latitudes and 35.5° to 38.3° E longitudes. The altitude of the Central Gondar zone ranges from 528 to 4620 meters above sea level (m.a.s.l). The average annual rainfall varies from 880 mm to 1772 mm, which is characterized by a monomodal type of distribution, with the temperature of 10°C to 44.5°C [29]. The West Gojjam zone is one of the zones in the Amhara region and lies between 36°30′ to 37°5′ E longitude and 10°16′ to 11°54′ N latitude. The total land area of the zone is 13,280 km^2^. Its elevation varies from 1500 to 3500 m.a.s.l. Most of the districts (75%) in the zone have ambient temperature ranging from 15 to 200°C, and the remaining (17%) have 20–270°C [30].

The South Gondar zone was encompassed in the study and is located in the Amhara region, 660 km northeast of Addis Ababa, Ethiopia. This zone is well known with a diverse topography ranging from flat and low grazing land to high cold mountains. The altitude is 1,500 to 3,600 m.a.s.l. The average yearly rainfall varies from 700 mm to 1300 mm, whereas the average daily temperature is 17°C [31].

### 2.2. Study Animals

The chickens in this study were existing exotic and local breeds comprising all groups, whose purpose ranged widely from breeders, egg production, and meat production in a farm from three selected zones. Moreover, chickens included in this study were healthy or diseased (cough, sneeze, and have tracheal rales for 10–14 days, conjunctivitis and dyspnea, and egg production may drop) and unvaccinated against IBV in the past. Since maternal antibody is expected to wane within three weeks of life, chickens less than three weeks of age were excluded from the study. For the farms, willingness of farmers to participate in the study was considered. The chickens were categorized into two age groups, namely, young (7 weeks) and adults (>8 weeks and above). Chickens having 3 weeks to 21 weeks of age were considered as young, whereas above 21 weeks of age were considered as adult. The age of chickens was determined from the owner response and according to Molla et al. [32].

### 2.3. Sample Size Determination

The sample size was determined by the formula described in [33] at 95% confidence interval and 5% of precision and considering that there was no such previous study in the study area (50%) in case of IBV. Thus, the minimum overall sample size required for the study was 384. However, to account for the design effect associated with the clustering of study units within flocks and locations, the sample size was multiplied by two resulting in a total sample size of 768 chickens.

The formula used was

(1) $$\begin{array}{c} n=\frac{z^{2}pq}{d^{2}},\\ n=\frac{1.96^{2}0.5\left(1-0.5\right)}{0.05^{2}}=384, \end{array}$$

where *n* is the total number of sample size and *z* = 1.96, *p* = 0.5, *q* = 1 − *p*, and *d* = 0.05 (the desired level of precession).

### 2.4. Blood Sample Collection and Serum Separation

Blood samples were collected aseptically from the wing vein of each chicken. About 2-3 ml of blood samples was collected using a sterile syringe with a 22-gauge needle. All necessary information related to each chicken including age, breed, sex, feeding status, farm type, production type, rearing method, house sanitation, litter management, house sanitation, ventilation type, zone, and farm shoe, and clothing was recorded on the data recording sheet by interviewing the owner and by observation during our visit in the farm. The blood samples in the syringe were allowed to clot in a slant position overnight to separate the sera. Subsequently, the sera were transferred into 1.5 ml Eppendorf tubes and kept at −20°C until the serological analysis for the presence of infectious bronchitis virus antibodies. The laboratory procedures were performed at the National Veterinary Institute (NVI), Serology Laboratory.

### 2.5. Study Design and Sampling Technique

A cross-sectional type of study design was employed from November 2020 to June 2021, with the aim to estimate the prevalence of infectious bronchitis virus in chicken and to identify associated risk factors. The sampling technique employed was multistage cluster sampling in which the district selection was done by the simple random method. Accordingly, a total of 7 districts, proportionally from each administrative zone (3 from Central Gondar, 2 from South Gondar, and 2 from West Gojjam), were selected. Again, proportional numbers of kebeles were selected by the simple random method from each district. Accordingly, 16 kebeles were considered in the study. Villages were also selected using simple random technique. The selection was facilitated by livestock experts and extension staff of the district. The simple random technique was used to sample individual chickens. A total of 768 chickens raised in the intensive and extensive production system, 256 from each respective zone, were selected.

### 2.6. Serological Analysis

Indirect ELISA (ID Screen® Infectious Bronchitis Virus Indirect) was used for the detection of antibodies against IBV in chicken sera according to the manufacturer's instructions. Unknown test samples were tested in parallel with positive and negative controls. All conditions were standardized according to kit manufacturer instructions using a precoated ELISA plate and ready-to-use reagents. The test was valid if the mean OD value of the positive control was greater than 0.250 and if the ratio of the mean values of the positive and negative control was greater than 3. The interpretation of the results was determined by the ELISA sample-to-positive (*S*/*P*) ratio for each serum. Those serum samples with an *S*/*P* value of greater than 0.2 were considered positive.

(2) $$\begin{array}{c} \frac{S}{P}\text{ ratio}=\frac{{\text{OD}}_{\text{sample}}-{\text{OD}}_{\text{NC}}}{{\text{OD}}_{\text{PC}}-{\text{OD}}_{\text{NC}}}, \end{array}$$

where *S*/*P* is the sample-to-positive ratio; OD is the optical density; ODNC is the optical density of negative control; and ODPC is the optical density of positive control.

### 2.7. Data Management and Analysis

The data collected from field level and laboratory investigation were coded into appropriate variables and entered into a Microsoft Office Excel 2019 spreadsheet. The data were checked for errors of entry, coded, and then imported to STATA for descriptive and further analyses. All statistical analyses were performed using STATA version 14 software. Descriptive statistics involving frequency and percentage was used to determine the seroprevalence of the disease. Binary logistic regression analysis was used to identify potential risk factors associated with IB. First, univariable logistic regression analysis with the flock as a random effect was performed, and potential risk factors (explanatory variables) with *p* values <0.25 were screened for the multivariable mixed-effect logistic regression. Association of environmental risk factors (breed, age, production purposes, farm type, rearing method, house sanitation, ventilation, disinfection of house, and farm shoes and clothing as a source of a pathogen) with seropositivity for IB was analyzed using multivariable mixed-effect logistic regression. The associations were considered statistically significant when *p* < 0.05 at 95% confidence level. Odds ratios at a 95% confidence interval were used to express the strength of the risk of the diseases associated with the tested factors.

### 2.8. Ethical Clearance

The research team members conducted the current study only after permitted ethical approval and statement given by the University of Gondar, Ethiopia. The current study was reviewed by the Institutional Ethical Review Board of the University of Gondar for its ethical soundness, and it was found to be ethically acceptable. Thus, the Research and Community Service Vice President Office awarded a grant under R. No. O/V/PRCS/05/495/2018.

## 3. Results

Serum samples of chickens from three zones of northwest Ethiopia were evaluated for previous exposure to infectious bronchitis virus using the indirect ELISA test. Of the total 768 blood sera tested, 184 samples were positive for infectious bronchitis virus antibody. The overall prevalence of IB was found to be 23.96% (95% CI: 20.98–27.14) (Table 1). Table 1 shows the seroprevalence of IB in three different zones, in which the highest seroprevalence was found in Central Gondar without significant difference among them (*p* > 0.05). The lowest seroprevalence was found in South Gondar.

Table 2 shows the analysis of host-related factors (breed, sex, production purposes, and age) in relation to the seroprevalence of IB. Accordingly, increased risk for IB was observed at young age and in layers as compared to adults (AOR: 0.07; 95% CI: 0.03–0.17) because immune cell and organ structures are immature in young chicken and in broilers (AOR: 0.31; 95% CI: 0.17–0.57) and dual (AOR: 0.006; 95% CI: 0.002–0.023) purposes, respectively. Broiler chickens selected for rapid growth are slower in degrading muscle proteins than layer hens selected for egg production. The rate of protein degradation in skeletal muscles of young layer hens is between around 1 and 9 times greater than that of broiler chickens. In layer chicks, excretion of NT methylhistidine derived from degradation of myofibrillar proteins is higher than in broiler chickens in relation to body weight and muscling. Unlike layer chickens, broiler chickens show low m-calpain activity and high calpastatin (calpain inhibitor) activity, which suggests that m-calpain and calpastatin activities in skeletal muscles differ between chicken types which have different rates of muscle growth. These results may suggest that leg muscles of layer hens compared to meat-type chickens are more susceptible to oxidative stress [34]. However, there was no significant relationship between sex and breed in relation to the occurrence of IB.

This study was also aimed at making a comprehensive study on the detection of antibody against IBV in chicken by considering environmental factor analysis namely, farm type, feeding status, rearing method, ventilation, house sanitation, litter management, disinfection of houses, carcass management, and farm shoe and clothing (Table 3). Accordingly, there is a significant relationship between farm type and seroprevalence of IB. Higher seroprevalence was found in intensive farm type as compared to the extensive type (AOR: 0.14: 95% CI: 0.06–0.34) with a significant difference of *p* < 0.001. Similarly, higher seroprevalence of IB was found in farms that used “all-in-all-out” operation than in farms that used different batches in one house operation (AOR: 0.16; 95% CI: 0.07–0.35) with a significant difference of *p* < 0.001. Besides, this study revealed higher seroprevalence of the IB in poorly ventilated farms (AOR: 4.77; 95% CI: 1.99–11.44), in farms which had no disinfection (AOR: 17.48; 95% CI: 6.87–44.46), in farms which did not remove used litters (AOR: 4.93: 95% CI: 1.77–13.75), and in farms which had no separate farm shoe and clothing (AOR: 2.98: 95% CI: 1.48–6.03) than their respective counterparts with a significant difference (*p* < 0.05).

**Figure 2:**
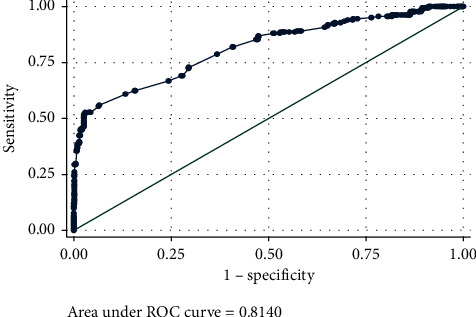
Receiver operator characteristic curve analysis and estimation of the study.

The Area under the ROC curve (AUC) is an effective way to summarize the overall diagnostic test. It takes values from 0 (perfectly inaccurate test) to 1 (perfectly accurate test). The AUC can be computed using the general rules; in these rules, the AUC value of 0.5 suggests no discrimination, 0.7–0.8 is considered acceptable, 0.8–0.9 is considered excellent, and more than 0.9 is considered outstanding [35]. Sensitivity versus 1–specificity plots were used for dichotomous outcome (positive/negative test result), which is called the receiver operating characteristic (ROC) curve, and AUC is an effective measure of the accuracy of meaningful interpretations [36] (Figure 2). Considering the obtained results of the current study, the AUC of the curve was 0.814. This suggested an 81.4% chance that the indirect ELISA test correctly distinguished infectious bronchitis virus-diseased chicken from nondiseased chicken based on the standard of the optical density value with an indirect ELISA reader. In this outcome, the results of sensitivity and specificity were 50.54% and 97.43%, respectively, and also, the correctly classified result was 86.20%.

## 4. Discussion

This work aimed to make a comprehensive study on the detection of antibodies against IBV in chicken by considering multifactor analysis. Consequently, a total of 768 samples were collected and tested for IBV by the indirect ELISA test, and an overall seroprevalence of IB was found to be 23.96% (95% CI: 20.98–27.14). IBV vaccine is not used in this study area; therefore, this is presumed to be a field infection. Even if apparent difference was noted, the present finding agrees with the report of Ramos et al. [37, 38] that demonstrated overall seroprevalence of IBV (25.53%) in industry-associated flocks of chickens inside Atlantic Biome forest, Northwestern São Paulo, Brazil. The results are also supported by the findings of Shettima et al. [39], who reported an overall prevalence of 26.6% from Nigeria. Moreover, this study finding was consistent with the finding of Bhuiyan et al. [40], who reported a prevalence of 23.82% in broilers, from Bangladesh, and Ayim-Akonor et al. [15], who revealed a prevalence of 21.2% in local chickens from Ghana.

However, this study finding was quite lower than the results of previous surveys in chicken, such as, studies by Hutton et al. [20], who reported a prevalence of 94.5% from the Ethiopian Institute of Agricultural Research (EIAR), located in Bishoftu, and Tesfaye et al. [21], who demonstrated four serotypes of infectious bronchitis virus with the prevalence of 70.60% from unvaccinated backyard and commercial farms in Ethiopia. This difference may attribute to the relatively high risk for chickens to get infection in and around Bishoftu, as described by Bekele et al. [41] that the disease has been speculated to be introduced concurrently with an increased number of commercial state and private poultry farms flourishing in the country especially in and around urban areas. Hence, Bishoftu and its surroundings represent the major and the principal site where a number of commercial farms are present and thus, chickens brought to this area will have higher risk of getting an infection from varied sources [32].

Similarly, this study finding was lower than the reports of Barberis et al. [42] from Algeria, Thekisoe et al. [43] from South Africa, Kouakou et al. [44] from Ivory-Coast, Benazir et al. [45] from Pakistan, Bhuiyan et al. [40], and Das et al. [46] from Bangladesh with a prevalence of 78.25%, 43%, 72%, 84.40%, 59.30%, and 79.38%, respectively. Besides, Owoade et al. [19], Emikpe et al. [47], and Mungadi et al. [48] revealed higher prevalence from Nigeria with a seroprevalence of 84%, 82.7%, and 89%, respectively. On the other hand, a lower seroprevalence (18.02%) was reported by Sabarinath et al. [49] in Grenada. The possible explanation of this difference could be due to use of different diagnostic test kits, sample sizes considered, the difference in agroclimatic conditions, and the distinctive farm management system.

Both local and exotic breeds exhibited antibodies to the virus, meaning that chickens from both breeds, regardless of their phenotypic and genotypic differences, faced the virus at some point. Tesfaye et al. [21] identified four serotypes of infectious bronchitis virus, namely, M41, D-274, 793B, and QX from unvaccinated backyard and commercial farms. Similarly, Tegegne et al. [22] detected IBV 793B (GI-13) strains in backyard flocks in Jimma Zone, with a prevalence of 6%. Besides, Hutton et al. [20] reported the detection of variant IBV (793B genotype) from the Ethiopian Institute of Agricultural Research (EIAR), located in Debre Zeit where no vaccination protocol has been implemented. As a result, these genotypes and serotypes may have arrived in the country from vaccinated animals elsewhere through importation of live chickens or migration of birds. Hence, considering these factors while studying IB epidemiology is important [50]. In addition, a higher prevalence (27.83%) was recorded in the exotic breed than in local breed (18.71%). This may be associated with low resistance to genetic diseases and other environmental stress in exotic breeds [51].

During the study, farm (management) type-wise analysis of the prevalence of IBV in chicken has indicated that intensive farming was slightly more infected than the extensive counterparts (Table 3) and the variation in susceptibility was statistically significant (*p* < 0.001). This finding was consistent with results observed by Shettima et al. [39] who reported higher seroprevalence in intensive farming with a significant difference. IB in intensive farming could be associated with the different types of management protocols. This could also have a relationship with either the presence of farms located in neighboring areas, since contact between poultry producing areas has been associated with the spread and maintenance of infectious diseases [50, 52].

Even though the seroprevalence of IB in the extensive farming is less than in its counterpart, i.e., intensive, having the prevalence of 20.77% is specifically alarming due to the highly transmissible nature of the disease and its capacity to spread to a substantial distance through aerosol and presence of carriers in the environment [48]. There is no use of IB vaccine in this study area. Besides, the density of chicken is low in the extensive farming system. Therefore, the virus has spread widely without the recognition of veterinary authorities. This has important implications to developing countries like Ethiopia where extensive chicken production accounts for the major portion of poultry population [21]. In addition, in this study area, chickens commonly raised in rural communities are mainly under the extensive management system where chickens have little or no veterinary care and scavenge for feed most of the day. This is the practice in most African and Asian countries, and this practice encourages easy spread of infectious agents [39].

Furthermore, the present study revealed that the seroprevalence of IB based on the production purposes in layer flocks was higher than that in broiler and dual flocks and the variation in susceptibility was statistically significant (*p* < 0.05). The present finding was compatible with Shettima et al. [39] who reported higher prevalence in layers with a significant difference. This could be ascribed to layer birds spending more time on the farm than others and subsequently becoming reinfected in the absence of a suitable control method [15]. This hypothesis is consistent with the explanation of Javed et al. [53] who revealed that the seroprevalence of IB increased when the chickens spend more time in the farm, because of the long period of exposure to the virus. Even if its difference was not significant, this study also revealed higher seroprevalence of IB in females (24.23%) than in males (23.29%). This result agrees with the report of Mungadi et al. [48] who revealed a higher prevalence in females than in males without significant differences in the study of local chickens in live bird markets in Sokoto State, Nigeria. This could be due to the fact that more females (549) were sampled than the males (219). This could be also associated with the presence of less-efficient immune response in males than in females due to the differences in the activity of humoral- and cell-mediated immune responses between the sexes [48].

There was a significant difference in the prevalence among chickens of different ages (*p* < 0.05). The higher prevalence was found in young chickens (32.12%) than in adults (21.22%). This finding was consistent with that of Mungadi et al. [48], who reported revealed a higher prevalence in growing ones than in adult chickens with a significant difference (*p* < 0.001). This finding was also consistent with the explanation of Cavanagh and Gelb [54], who stated that even though all age groups of chicks are susceptible to IBV, young chicks are more susceptible than older ones. In addition, as the age of chicken advances, resistance to a disease also increases.

Cavanagh and Naqi [55] and Emikpe et al. [47] reported that maternal antibodies decline rapidly and early within the 3rd week of age. In contrary, in this study, the sampled chickens were three and above weeks of age. It means that after the decline of maternal antibodies, a positive result to serological tests occurs following exposure to a wild virus. Consequently, antibodies detected in adult chickens could be considered as an indicator of infection with wild-type viruses [42].

Among the environmental risk factors, rearing method, ventilation, house sanitation, disinfection of houses, and farm shoe and clothing were statistically significant (*p* < 0.05, Table 3). This finding was consistent with the explanation of Dhama et al. [18], who stated that good management practices are important to prevent this disease, such as strict isolation/quarantine, restocking/repopulation with disease-free day-old chicks, and adapting appropriate cleaning, disinfection, and hygienic measures in the poultry farm. In addition, to minimize the intensity of infectious virus and limit its introduction in poultry house, controlled visitors' access to the farm premises, keeping separate clothing, footwear, and equipment for each farm/unit, controlling movements of farmworkers/personnel and equipment between farms, and keeping appropriate footbaths with disinfectants at the entry points are important.

## 5. Conclusion and Recommendations

In conclusion, this preliminary study had revealed an overall seroprevalence of 23.96% among chickens in northwest Ethiopia. The seroprevalence finding in the present study indicated that the organism is circulating among the population of chickens and high enough to cause significant economic losses, limiting the productivity of chickens in the study area. No vaccination against IB is carried out in the study area; the seroprevalence observed may suggest a natural infection. This study also indicated that seropositivity for IBV (occurrence of IB disease) was significantly associated with age, farm type, production purposes, rearing method, ventilation type, house sanitation, disinfection of the house, and farm shoe and clothing. These host and environmental variations need to be taken into account while scheming prevention and control actions for IB.

Based on the above conclusion, the following recommendations are forwarded:

- Poultry houses should be cleaned and disinfected
- Poultry houses should be well ventilated
- The farm attendants should have separate farm shoe and clothing
- Further studies on the virus isolation and molecular characterization of the target gene are needed in the study area

## Figures and Tables

**Table 1:** Seroprevalence of IB in three different zones.

Variables	Locations	No. of examined samples	No. of positive samples	Prevalence % (95% CI)	Univariable COR (95% CI)	*p* value
Zone	Central Gondar	256	63	24.61 (19.46–30.36)	Reference	
	South Gondar	256	59	23.05 (18.03–28.70)	0.92 (0.61–1.38)	0.678
	West Gojjam	256	62	24.22 (19.10–29.94)	0.98 (0.65–1.47)	0.918

	Total	768	184	23.96% (95% CI: 20.98–27.14)		

COR: crude odds ratio; 95% CI: 95% confidence interval. *p* values <0.05 were statistically significant, and *p* values <0.001 were strongly significant.

**Table 2:** Univariable and multivariable mixed-effect logistic regression analysis of host potential risk factors for infectious bronchitis virus.

Variables	Category	No. of examined samples	No. of positive samples	Prevalence % (95% CI)	Univariable COR (95% CI)	*p* value	Multivariable AOR (95% CI)	*p* value
Breed	Local	326	61	18.71 (14.63–23.38)	Reference			
	Exotic	442	123	27.83 (23.70–32.26)	1.68 (1.18–2.37)	0.004	1.33 (0.84–2.11)	0.217

Sex	Male	219	51	23.29 (17.86–29.46)	Reference			
	Female	549	133	24.23 (20.70–28.03)	1.05 (0.73–1.52)	0.783		

Production purposes	Layers	393	112	28.50 (24.09–33.24)	Reference			
	Broiler	193	38	19.69 (14.33–26.01)	0.62 (0.41–0.93)	0.022	0.31 (0.17–0.57)	<0.001
	Dual	182	34	18.68 (13.30–25.11)	0.58 (0.37–0.89)	0.012	0.006 (0.002–0.023)	<0.001

Age	Young	193	62	32.12 (25.60–39.21)	Reference			
	Adult	575	122	21.22 (17.94–24.79)	0.57 (0.40–0.82)	0.002	0.07 (0.03–0.17)	<0.001

COR: crude odds ratio; AOR: adjusted odds ratio; 95% CI: 95% confidence interval. *p* values <0.05 were statistically significant, and *p* values <0.001 were strongly significant.

**Table 3:** Univariable and multivariable mixed-effect logistic regression analysis of environmental risk factors for infectious bronchitis virus.

Variables	Category	No. of examined samples	No. of positive samples	Prevalence % (95% CI)	Univariable COR (95% CI)	*p* value	Multivariable AOR (95% CI)	*p* value
Ventilation	Good	585	121	20.68 (17.47–24.20)	Reference			
	Poor	183	63	34.43 (27.57–41.79)	2.01 (1.40–2.90)	<0.001	4.77 (1.99–11.44)	<0.001

House sanitation (used litter)	Completely disposed	78	7	8.97 (3.69–17.62)	Reference			
	Partially disposed	386	97	25.13 (20.88–29.77)	3.40 (1.52–7.65)	0.003	4.19 (1.54–11.40)	0.005
	Not at all	304	80	26.32 (21.45–31.65)	3.62 (1.60–8.20)	0.002	4.93 (1.77–13.75)	0.002

Disinfection of the house	Disinfection	178	30	16.85 (11.67–23.18)	Reference			
	No disinfection	590	154	26.10 (22.60–29.84)	1.74 (1.13–2.69)	0.012	17.48 (6.87–44.46)	<0.001

Carcass management	Buried or burning	168	37	22.02 (16.01–29.06)	Reference			
	Throwing to nearby space	600	147	24.5 (21.11–28.15)	1.15 (0.76–1.73)	0.507		

Farm shoe and clothing	Have	131	20	15.27 (9.58–22.59)	Reference			
	Do not have	637	164	25.75 (22.39–29.33)	1.92 (1.16–3.20)	0.012	2.98 (1.48–6.03)	0.002

Farm type	Intensive	219	70	31.96 (25.84–38.58)	Reference			
	Extensive	549	114	20.77 (17.45–24.40)	0.75 (0.63–0.89)	0.001	0.14 (0.06–0.34)	<0.001

Rearing method	All-in-all-out	157	59	37.58 (29.99–45.65)	Reference			
	Different batches in one house	611	125	20.46 (17.33–23.88)	0.43 (0.29–0.62)	<0.001	0.16 (0.07–0.35)	<0.001

Feeding status	Properly fed	173	37	21.39 (15.53–28.25)	Reference			
	Underfed	595	147	24.71 (21.29–28.38)	1.21 (0.80–1.81)	0.369		

Litter management	Buried	25	4	16 (4.54–36.08)	Reference			
	Used as fertilizer	570	135	23.68 (20.25–27.40)	1.63 (0.55–4.83)	0.379		
	Accumulate to the nearby free space	173	45	26.01 (19.65–33.22)	1.85 (0.60–5.67)	0.284		

COR: crude odds ratio; AOR: adjusted odds ratio; 95% CI: 95% confidence interval. *p* values < 0.05 were statistically significant, and *p* values <0.001 were strongly significant.

## Data Availability

Data will be made available upon request to the primary author.
